# Quality of life after Supracricoid Partial Laryngectomy

**DOI:** 10.1186/s40463-021-00499-w

**Published:** 2021-03-25

**Authors:** Marianne Yumi Nakai, Marcelo Benedito Menezes, Julia Vilas Boas Gonçalves de Carvalho, Lucas Porto Maurity Dias, Leandro Augusto de Barros Silva, Lucas Ribeiro Tenório, Antonio José Gonçalves

**Affiliations:** 1 Santa Casa de São Paulo School of Medical Sciences and Irmandade da Santa Casa de Misericórdia de São Paulo, Rua Cesário Motta Jr 112, São Paulo, SP Brazil; 2 Santa Casa de São Paulo School of Medical Sciences, Sao Paulo, Brazil; 3 Irmandade da Santa Casa de Misericórdia de São Paulo, Sao Paulo, Sao Paulo Brazil

## Abstract

**Background:**

Supracricoid partial laryngectomy has good oncologic results in the treatment of advanced laryngeal cancer with the advantage of preserving larynx phonatory function when compared with total laryngectomy. However the rehabilitation could be a challenge, especially regarding swallowing function. Is supracricoid partial laryngectomy associated with better quality of life than total laryngectomy?

**Methods:**

Survey study that included 33 patients (16 total laryngectomy and 17 supracricoid partial laryngectomy) with advanced larynx cancer surgically treated and fully rehabilitated. The quality of life were evaluated with EORTC QLQ C30 and H&N 35 instrument.

**Results:**

Patients who underwent supracricoid partial laryngectomy obtained better scores in global health status-quality of life and general activities and had lower levels of sensory and speech-related symptoms.

**Conclusion:**

SPL was associated with better quality of life when compared with TL.

**Graphical abstract:**

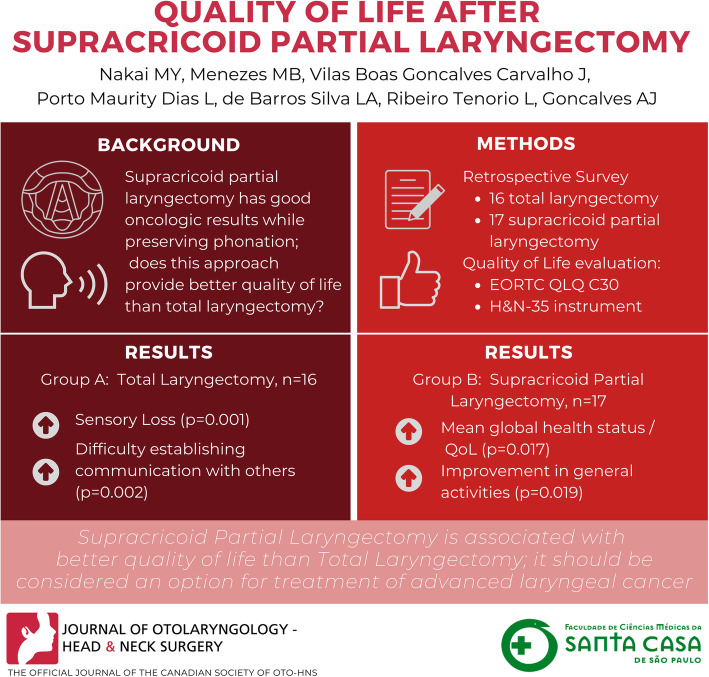

**Supplementary Information:**

The online version contains supplementary material available at 10.1186/s40463-021-00499-w.

## Introduction

Laryngeal cancer is one of the most common tumors in the head and neck region, accounting for approximately 25% of malignancies affecting this area and 2% of all malignancies [1].

The introduction of new surgical techniques and the availability of detailed information about laryngeal anatomy have enabled great advances in the treatment of laryngeal cancer, thereby increasing treatment effectiveness and allowing greater preservation of the functions of this organ. Currently, laryngeal cancer is a disease with a high cure rate in the early stages and a good rate of local control in more advanced cases [2].

The larynx is responsible for three important functions in the human body: swallowing, breathing, and phonation. Patients with laryngeal cancer, even after undergoing treatment, usually present impairments in these functions, which results in a significant change in their quality of life. There is a growing concern about reducing the impact of treatment on the quality of life of these patients without compromising their prognosis. Therefore, partial laryngectomy techniques have been the subject of ongoing study, and their use has become increasingly more comprehensive.

Supracricoid horizontal partial laryngectomy (SPL) was described by Piquet et al., who relied on a technique developed by Majer and Rieder in 1959. However, it was only in the 1990s that Laccourreye et al. popularized the use of SPL for the treatment of glottic cancer in France and throughout Europe. Nevertheless, the technique has only become popular in the United States in recent years. This technique has also been adapted for the treatment of supraglottic and hypopharyngeal cancers. Serial studies have demonstrated that SPL is a viable alternative to total laryngectomy (TL) and a valid surgical rescue option in cases of unsuccessful radiation therapy [3].

The technique consists of the resection of the entire thyroid cartilage from the cricoid to the base of the epiglottis while preserving one or preferably two arytenoids with intact mobility and innervation, which allows the removal of the entire paraglottic spaces and avoids the opening of the larynx close to the tumor. The epiglottis may or may not be resected, depending on the extent of the tumor. The major laryngeal defect is reconstructed through cricohyoidoepiglottopexy or cricohyoidopexy (when the epiglottis is included in the resection), which consists of the fixation of the cricoid in the hyoid bone [4]. The preservation of the arytenoids allows speech rehabilitation, and the preservation of the cricoid allows the decannulation of the tracheostomy.

According to the WHO, quality of life is an individual’s perception of his/her position in life in the context of the culture and value system in which he/she exists and in relation to his/her goals, expectations, standards, and concerns. It is a concept of comprehensive scope that is affected in a complex way by the individual’s physical health, psychological state, level of independence, social relations, and relations with the environment [5].

Until the late 1980s, cancer treatment aimed at maximizing patient survival, with little emphasis on psychosocial aspects. The treatment’s impact was assessed primarily in terms of the patient’s biological data, such as tumor size, staging, local growth control, and survival. There was little concern about the effects of treatment on the patient’s quality of life. However, in recent years, interest in the psychosocial aspects of patients has increased, and the patient’s functional rehabilitation has become an important factor in the treatment of cancer.

There is no clear consensus about which factors to evaluate and how to evaluate the factors that make up quality of life. Nevertheless, it is accepted that at least four aspects should be considered: physical complaints, treatment effects, social interaction, and psychological and functional status [5–8].

Currently, several questionnaires aim to obtain an adequate evaluation of the patients’ quality of life in different ways. Nevertheless, there is a consensus that a combination of generic questionnaires with specific questionnaires allows a broader and more reliable view of the patient’s real-life situation [8, 9].

In 1992, Kaasa published an essay on the questionnaires that were available for assessing quality of life in the early 1990s. In his study, the EORTC QLQ-C30 questionnaire was cited as a relevant instrument for adequately assessing quality of life in cancer patients [10]. This questionnaire is multidimensional and has been cross-culturally validated, demonstrating similar psychometric properties when applied in different languages and countries. Its association with the H&N35, which is a specific questionnaire, allows a good balance between generality and specificity. Moreover, it is easily self-administered [10, 11].

Head and neck cancers deserve special attention when considering life quality. They usually have a very visible location and involve functions that play a fundamental role in the individual’s social life. In addition, the impact of treatment is often perceived only after treatment is completed.

Assessing perceived health from the patient’s point of view allows the physician to choose a treatment that not only targets survival but also meets the patient’s psychosocial needs. This ensures a better quality of life and, consequently, greater patient satisfaction with treatment. For this to be possible, it is important to provide multidisciplinary care that includes the surgeon, oncologist, psychiatrist, speech therapist, and other health professionals [12].

Although the literature on the quality of life of patients with cancer has been enriched in recent years, there is still a lack of studies on the quality of life of patients undergoing laryngectomy. Many studies touch on the theme, dealing with more prevalent specific symptoms, such as phonation and deglutition. However, only a few studies address the quality of life of patients in terms of broader symptoms and with larger study samples [13].

## Objective

To evaluate the quality of life of patients with laryngeal cancer who have undergone surgical treatment with SPL or TL in the late postoperative period.

### Patients and methods

This survey included 33 patients with laryngeal cancer who underwent laryngectomy. The following inclusion and exclusion criteria were used.

#### Inclusion criteria

Patients with clinical T3 stage cancer who underwent treatment via SPL or TL:

- Age greater than 18 years
- Postoperative period of at least 6 months
- Full rehabilitation: swallowing, speech and respiratory therapy

#### Exclusion criteria

- Presence of locoregional metastasis or recurrence
- Severe mental deficiency, dementia, or psychosis
- Severe auditory deficiency
- Neurological disease causing a swallowing disorder
- Alcoholism
- Inability to understand the instructions and objectives of the present study

All patients were duly informed about the research and agreed to participate by signing the informed consent form.

Rehabilitation was evaluated by a speech therapist specializing in head and neck surgery, and all the included patients were considered completely rehabilitated: no feeding tube or tracheostomy was required, and the patients were considered good speakers in terms of a phonatory evaluation. In the TL group, all of the included patients underwent voice rehabilitation with the esophageal voice method.

Quality of life was assessed using a self-administered questionnaire developed by the Quality of Life Study Group of the European Organization for Research and Therapy of Cancer [7]. The protocol was cross-culturally validated and consisted of 30 generalized questions (QC-30 version 3.0) and 35 specific questions related to head and neck symptoms. The questionnaire was designed to assess physical functioning, role functioning, cognitive functioning, emotional functioning, social functioning, pain, fatigue, emesis, and global health status/QoL using multi-item scales [10, 11].

A demographic data sheet was used to obtain data on age, sex, schooling, occupation, and information related to the disease and treatment (Additional file 1).

The patients were contacted by telephone and invited to come to the hospital to participate in a private interview of variable length, in which the abovementioned questionnaires were asked in the presence of an appropriately trained medical student under the supervision of a trained physician.

The patients were divided into two groups according to the type of surgery they underwent for the treatment of laryngeal cancer:

- Group A: patients who underwent TL
- Group B: patients who underwent SPL

### Data analysis

Sociodemographic and EORTC QLQ-C30 data were coded and entered into a database created using IBM SPSS Statistics for Macintosh, Version 21.0 (IBM Corp., Armonk, NY, USA).

The responses obtained from the EORTC QLQ-C30 questionnaire were used to generate a score representing the quality of life for each patient. This score was calculated according to the instructions contained in the EORTC QLQ-C30 Scoring Manual [7, 14].

The association among variables obtained from the sociodemographic record and the type of surgery was evaluated using the chi-square test. Numerical variables were compared between groups using the Mann-Whitney nonparametric comparison test.

The scores obtained using the EORTC QLQ-C30 and H&N35 questionnaires were analyzed using the Mann-Whitney nonparametric comparison test to evaluate significant differences between the groups.

## Results

Group A had 16 patients: 1 woman (6.2%) and 15 men (93.7%). The mean age was 56 years. Most of the patients (68.7%) had a low level of education (i.e., they were illiterate or had not completed primary school), 50% were married, 81.2% were Catholic, 43.7% were unemployed and receiving disability benefits, 75% lived with family, and 56.2% took more than a year to seek medical help after the onset of symptoms. In this group, 6 patients (37.5%) underwent radiotherapy after surgery. Eight patients were node negative (50%). The average time between the surgery and the interview was 20.1 months (Table 1).

**Table 1:** Description of measures categorized by type of surgery and outcome of sociodemographic questionnaire, radiotherapy and time elapsed since surgery at time of survey

Variable	Category	TL		SPL		Total		p
		no.	%	no.	%	no.	%	
Age range (Years)	31–40	0	0	1	5.9	1	3	**0.5**
	41–50	5	31.2	2	11.7	7	21.2	
	51–60	5	31.2	6	35.3	11	33.4	
	61–70	4	25	7	41.2	11	33.4	
	71+	2	12.5	1	5.9	3	9	
Schooling	Primary school incomplete	6	37.5	8	47.0	14	42.4	**0.6**
	Primary school complete	1	6.2	3	17.6	4	12.2	
	High school incomplete	1	6.2	2	11.9	3	9	
	High school complete	2	12.5	1	5.9	3	9	
	Higher education incomplete	1	6.2	0	0	1	3	
Civil status	Married	8	50	12	70.6	20	60.6	**0.4**
	Single	4	25	2	11.8	6	18.2	
	Divorced	2	12.5	2	11.8	4	12.1	
	Stable union	2	12.5	0	0.0	2	6	
	Widower	0	0.0	1	5.9	1	3	
Religion	None	2	12.5	1	5.9	3	9	**0.2**
	Catholic	13	81.2	11	64.7	24	72.7	
	Evangelical	0	0.0	4	23.5	4	12.1	
	Spiritist	0	0.0	1	5.9	1	3	
	Other	1	6.2	0	0	1	3	
Professional status	Work at home	1	6.2	0	0	1	3	**0.3**
	Work outside	2	12.5	4	23.6	6	18.2	
	Does not work (sick pay)	7	43.7	3	17.6	10	30.3	
	Retired	4	25	9	52.9	13	39.4	
	Other statuses	2	12.5	1	5.9	3	9	
Housing	Alone	2	12.5	1	5.9	3	9	**0.4**
	With current family	12	75	16	94.1	28	84.8	
	With original family	1	6.2	0	0	1	3	
	Others	1	6.2	0	0	1	3	
Time to seek medical help	Never suspected	0	0	0	0	0	0	**0.2**
	< 1 month	1	6.2	0	0	1	3	
	1 to 3 months	1	6.2	3	17.6	4	12.1	
	3 to 6 months	4	25	8	47.0	12	36.4	
	More than 6 months	1	6.2	2	11.8	3	9	
	> 1 year	9	56.2	4	23.6	13	39.4	
Radiotherapy after surgery	Yes	5	31.2	7	41.2	12	36.3	**0.4**
	No	11	68.7	10	58.8	21	63.6	
Avarage time: surgery – survey (Mounths)		20.2		26				**0.9**
Avarage hospitalization time (Days)		13		10.1				**0.08**
Complication		6	35.2	6	37.5			**0.1**

Group B had 17 patients: 6 women (35.3%) and 11 men (64.7%). The mean age was 58 years. The majority (47%) had not completed primary school, 70.6% were married, 64.7% were Catholic, and 53% were retired. Forty-seven percent of the patients took 3 to 6 months to seek medical help after the onset of symptoms. Ninety-four percent of the patients reported living with their family. In this group, 7 patients (41.1%) underwent radiotherapy after surgery. Twelve patients were node negative (70.5%). The average time between the surgery and the interview was 26 months (Table 2).

**Table 2:** Results of overall life quality—EORTC QLQ-C30

		Surgery			
EORTC QLQ-C30		SPL^b^	TL^b^	Reference value^**a**^	p
**Global health status/QOL**	–	87.75	73.44	66.7	**0.017**
**Functioning**	Physical	87.06	83.75	73.3	**0.465**
	Role	92.16	65.63	83.3	**0.019**
	Emotional	69.12	54.17	75	**0.127**
	Cognitive	91.18	86.46	83.3	**0.423**
	Social	92.16	81.25	83.3	**0.146**
	**Mean**	**86.57**	**74.11**	**77.48**	**0.236**
**Symptoms**	Dyspnea	21.57	31.25	33.3	**0.510**
	Insomnia	19.61	18.75	0	**0.986**
	Fatigue	9.15	22.92	33.3	**0.444**
	Loss of appetite	13.73	14.58	0	**0.790**
	Nausea and vomiting	3.92	6.25	0	**0.557**
	Constipation	9.80	22.92	0	**0.606**
	Diarrhea	0.00	8.33	0	**0.557**
	Pain	14.71	27.08	16.7	**0.402**
	Financial difficulty	11.76	31.25	0	**0.245**
	**Mean**	**11.58**	**20.37**	**10.41**	**0.566**

^a^ reference values were obtained according to EORTC reference value [15]

^b^the quality of life was measured by a scale 0 to 100 in witch higher scores means better quality of life

Data from the sociodemographic questionnaire did not exhibit significant differences between the two groups (Table 1).

Additionally, no significant differences were noted between the two groups regarding the time elapsed since surgery at the time of the survey with a *p*-value of 0.8 (Table 1).

None of the TL patients were previously SPL patients.

Regarding the evaluation of quality of life, the mean global health status/QoL was significantly higher in patients who underwent SPL compared with those who underwent TL (*p* = 0.017) (Table 2).

When each functional field of the EORTC QLQ-C30 was analyzed individually, a significant gain in general activities (role functioning) was observed by these patients (*p* = 0.019) (Table 2).

The other functional domains measured in the EORTC QLQ-C30 did not present statistically significant differences (Table 2).

In the scale that evaluated specific symptoms and individual items on the EORTC QLQ-C30, no significant difference was observed between the groups (Table 3).

**Table 3:** Results of life quality specific for head and neck—EORTC H&N35

	Surgery		
EORTC QLQ H&N35	SPL	TL	p
Pain	4.90	10.93	**0.901**
Swallow	14.70	9.37	**0.657**
Tooth	9.80	0.00	**0.588**
Open the mouth	5.88	8.33	**0.763**
Dry mouth	23.53	31.25	**0.533**
Sticky saliva	41.17	29.16	**0.382**
Sensitive	2.94	38.54	**0.001**
Cough	31.37	37.50	**0.790**
Feeling sick	5.88	10.41	**0.557**
Eat socially	3.43	9.89	**0.606**
Communication	35.29	62.50	**0.002**
Social contact	8.23	15.00	**0.081**
Loss of sexuality	27.45	22.91	**0.631**
Use of analgesics	41.17	18.75	**0.276**
Use of supplements	35.29	12.50	**0.276**
Use of probe	11.76	6.25	**0.790**
Weight loss	5.88	0.00	**0.790**
Weight gain	41.17	68.75	**0.179**
**Mean**	**19.43**	**21.78**	**0.488**

In the specific questionnaire for the evaluation of head and neck symptoms (the H&N35), we observed that patients who underwent TL had greater sensory loss than those who underwent SPL (*p* = 0.001) (Table 3).

In addition, on the H&N35 questionnaire, we observed that patients who underwent TL presented greater difficulties establishing communication with other individuals than those who underwent SPL (*p* = 0.002) (Table 3).

Several parameters were better in patients who underwent SPL than in those who underwent TL, but the differences were not significant (Tables 2 and 3).

The mean length of stay was 10.1 days in the SPL group and 13 days in the TL group. No significant difference was noted between the two groups (*p* = 0.085) (Table 1).

The overall complication rate was 42.4%. In both groups, 6 patients presented complications, corresponding to 35.2% in the SPL group and 37.5% in the TL group (Table 1).

The complications in the SPL group mostly included small tracheocutaneous fistulas that were surgically corrected, and the patients maintained good rehabilitation. In the TL group, three patients underwent surgery for tracheostoma enlargement, and three presented pharyngocutaneous fistulas, one of whom underwent surgical correction of this fistula, which increased the hospitalization time. At the time of the interview, all these patients experienced complete resolution of their complications and were considered fully rehabilitated (in terms of phonation and swallowing) (Table 1).

## Discussion

The mean global health status/QoL was significantly increased in patients who underwent SPL compared with those who underwent TL (87 vs 73/*p* = 0.017). The most impaired domain in the TL group was emotional. However, compared with the SPL group, role functioning showed the greatest loss (92 vs 65/*p* = 0.019).

When we compared the scores obtained using EORTC reference values for laryngeal and hypopharyngeal cancers, our patients had higher general QoL scores in both groups [15].

Patients in the TL group had a significantly higher score for sensory symptoms on the specific questionnaire (EORTC H&N35) than the SPL group (2.9 vs 38/*p* = 0.001) likely because TL stops the airflow through the mouth, nose, and pharynx, thereby greatly impairing the patient’s senses of smell and taste.

Voice outcomes are comparable between patients who undergo TL and SPL in most studies [16]. In Brazil, however, since most patients use the public health system, the esophageal voice is the most commonly applied rehabilitation technique. Only 30% of patients achieve success with speech therapist training; this can cause communication difficulty, which makes it difficult to adapt to and interact in the work environment. In addition, there is an aesthetic aspect; i.e., TL introduces the stigma of a definitive tracheostomy in laryngectomized patients, which may affect the patient’s acceptance in their living environment both at work and in public places. The patient’s self-image can become impaired by these factors, which discourages his/her search for pleasurable activities. This notion may explain the low values for overall quality of life and the role functioning domain in the TL group.

Our study included patients who underwent surgery from 1991 to 2016. During this period, the hospital switched medical records from physical paper to electronic records, which added difficulty in gathering the data and recovering missing information. We acquired quality of life information during a single postoperative period. The quality of life status before surgery and the changes over time were not measured. Thus, it is possible that some bias occurred, and we cannot be sure whether the surgery was the only factor responsible for the differences in quality of life between groups.

We chose to include only completely rehabilitated patients because swallowing function is impaired after SPL – even in completely rehabilitated patients. In addition, after proper intervention, the rate of complete rehabilitation is high (80–90%) [17, 18]. In contrast, the swallowing function of TL patients is almost normal even without any intervention. Regarding oncologic control, the effectiveness of both techniques is similar [19–21]; however, some surgeons still defend against TL surgery in the treatment of advanced laryngeal carcinoma mainly due to swallowing impairment and difficulties in rehabilitation.

The most important symptom in the TL group was speech difficulty. This finding is consistent with data in the literature, which point to communication as the most impaired function after TL [22, 23].

The most important symptom presented in the SPL group was sticky saliva. Sticky saliva is probably due to radiation therapy, which often produces this symptom. In fact, the high symptomatic score for sticky saliva in the SPL group occurred mainly due to a decrease in other symptoms and not because of an increase in sticky saliva symptoms. On average, the time before seeking medical help was lower in the SPL group (3 to 6 months) compared with the TL group (> 1 year); however, this difference was not statistically significant (*p* = 0.234). This finding indicates greater health-related self-care, which may influence a greater search for quality of life. This may be reflected in the gains observed in practically all the evaluated domains. In addition, the patients included in our study exhibited 100% adherence to the rehabilitation treatment and were therefore able to completely benefit from this type of surgery (e.g., through better vocal quality and the absence of a tracheostomy). These factors certainly influenced the overall improvement in quality of life, which presented a statistically significant better upper value in the SPL group compared with the TL group (*p* = 0.017).

Complete rehabilitation of patients who undergo TL or SPL requires the understanding, collaboration, and compliance of the patient. In addition, the presence of a trained multidisciplinary team is indispensable.

SPL facilitates voice rehabilitation. The speech rehabilitation process is quite natural, and tracheostomy is temporary, which preserves the patient’s appearance [17, 18, 21, 24]. However, swallowing rehabilitation requires hard work with a specialized team and a multidisciplinary approach (involving the surgeon, speech therapist and psychologist) and depends on the patient’s adherence and his/her cognitive ability to understand and assimilate the commands necessary to perform swallowing maneuvers and exercises [18].

TL does not greatly modify the swallowing mechanisms. After the pharyngeal suture heals, the majority of patients can eat normally without any training. Voice is the most impaired function in TL patients, and rehabilitation can be a challenge [23]. It is important to emphasize that all TL patients included in this study were considered good speakers based on evaluation by a speech therapist who specialized in minimizing the impact of voice rehabilitation on quality of life. However, voice rehabilitation was achieved with the esophageal voice method. This could be a source of bias in the quality of life results for TL patients because the first choice for voice rehabilitation is a tracheal esophageal prosthesis, which provides a longer phonation time than esophageal voice. Another important issue in TL patients is definitive tracheostomy, which can result in social stigma, especially because these devices are rarely covered by clothes.

Patients who undergo SPL and do not achieve successful swallowing rehabilitation need to undergo TL because they are dependent on a feeding tube [25]. However, patients who undergo TL and do not rehabilitate can typically find other ways of communicating (writing, gesticulation, articulated speech, etc.). None of our TL patients were previous SPL patients.

Despite the difficulties mentioned above, the patients in this study who underwent SPL presented a higher quality of life than those who underwent TL, obtaining statistically significantly better scores for general QoL and the overall domains. Symptoms related to swallowing (questions 5 to 8 of EORTC H&N35) did not present statistically significant differences between groups. These results demonstrate that despite the abovementioned difficulties, swallowing is not a factor that negatively influences the quality of life of patients who undergo rehabilitation after SPL.

## Conclusion

SPL is associated with better quality of life than TL and should be considered an option for the treatment of advanced laryngeal cancer despite swallowing rehabilitation difficulties.

## Supplementary Information

**Additional file 1.**

## Data Availability

The datasets used and analysed during the current study are available from the corresponding author on reasonable request.
