# Supplementary material for: Quality of life after Supracricoid Partial Laryngectomy
Source: J Otolaryngol Head Neck Surg. 2021 Mar 25;50:20. doi: 10.1186/s40463-021-00499-w (PMC7995799; doi:10.1186/s40463-021-00499-w)
Supplement: Supplementary file 1 — Additional file 1. [file 40463_2021_499_MOESM1_ESM.docx]

**Appendix I**

DEMOGRAPHIC QUESTIONNAIRE

Name: ___________________________________________ Date: _______________

1. Age:

( ) 18– 30 years ( ) 51 – 60 years

( ) 31 – 40 years ( ) 61 – 70 years

( ) 41 – 50 years ( ) + 71 years

2. Schooling:

( ) Illiterate

( ) Elementary School ( ) complete ( ) incomplete

( ) High School ( ) complete ( ) incomplete

( ) College ( ) complete ( ) incomplete

3. Marital Status

( ) Married ( ) Divorced

( ) Single ( ) Commom-law marriage

( ) Separated ( ) Widower

4. Do you have any kids?

( ) No

( ) Yes – How many?___________ Kid’s ages:____________________

5. Religion:

Do you practice it?: ( ) Y ( ) N

( ) No ( ) Buddhist

( ) Catholic ( ) Spiritist

( ) Evangelic ( ) Other:_____________

6. Professional Status:

( ) Home office

Which one?:__________________________________

( ) Standard office

Which one?:__________________________________

( ) Do not work – welfare aid

( ) Retired

( ) Other: ____________________________________________

7. Living Status:

( ) Alone ( ) With friends

( ) With spouse and children ( ) Other: ________________

( ) With your parents

8. How long did you take to look for medical help after starting to feel the symptons?

( ) Less than a month ( ) over 6 months

( ) 1 to 3 months ( ) over a year

( ) 3 to 6 months ( ) Never suspected
